# MUC14-Related ncRNA-mRNA Network in Breast Cancer

**DOI:** 10.3390/genes12111677

**Published:** 2021-10-23

**Authors:** Shuqian Wang, Jing Jin, Jing Chen, Weiyang Lou

**Affiliations:** 1 Department of Breast Surgery, The First Affiliated Hospital, School of Medicine, Zhejiang University, Hangzhou 310003, China; wangshuqian@zju.edu.cn; 2 Department of Neurosurgery, The First Affiliated Hospital, School of Medicine, Zhejiang University, Hangzhou 310003, China; 1509008@zju.edu.cn; 3 Department of Oncology, The First Affiliated Hospital of Jiaxing University, Jiaxing 314000, China

**Keywords:** mucin 14 (MUC14), noncoding RNA (ncRNA), microRNA (miRNA), breast cancer, bioinformatic analysis

## Abstract

**Abstract**: Background Growing evidences have showed that mucins (MUCs) are linked to occurrence and progression of human cancers. However, a comprehensive study regarding the expression, diagnosis, prognosis and mechanism of MUCs in breast cancer remains absent. **Methods:** A series of in silico analyses were employed in this study. **Results:** After performing comprehensive analysis for MUCs, MUC14 was identified as the most potential regulator in breast cancer, with downregulated expression in both mRNA and protein levels and significant diagnostic and prognostic values in breast cancer. Mechanistic exploration revealed that a potential ncRNA-mRNA axis, involving LINC01128/LINC01140/SGMS1-AS1/LINC00667-miR-137/miR-429-BCL2, might be partially responsible for MUC14′s functions in breast cancer. **Conclusions:** Collectively, our study elucidated a key role of MUC14 in breast cancer and also provided some clues for explanation of the molecular action mechanism of MUC14 in breast cancer.

## 1. Introduction

Breast cancer, the most frequent cancer type in women worldwide, is one of the leading causes of cancer-related deaths in females [1,2]. According to molecular expression characteristics, breast cancer is generally classified into luminal A breast cancer, luminal B breast cancer, Her2 positive breast cancer and triple negative breast cancer (TNBC), among which TNBC is the most aggressive type [3]. Currently, treatment regimens for breast cancer contain surgical resection, chemotherapy, radiotherapy, endocrine therapy and molecular target therapy [4]. Despite great advances have been achieved in diagnosis and treatment of breast cancer, the prognosis of patients, especially TNBC, remains dismal and unsatisfactory [5]. It is urgent and meaningful to identify and develop promising therapeutic targets and diagnostic and prognostic biomarkers, which is important for the improvement of the therapeutic effects and the accuracy of diagnostic and prognostic prediction.

Mucins (MUCs), a group of large *O*-glycoproteins, are generally divided into three subgroups, including membrane-bound MUCs (consisting of MUC1, MUC3A, MUC4, MUC12, MUC13, MUC15, MUC16, MUC17, MUC20, MUC21 and MUC22), secreted MUCs (consisting of MUC2, MUC5AC, MUC5B, MUC6, MUC7, MUC9/OVGP1 and MUC19) and atypical endothelial MUCs (MUC14/EMCN and MUC18/MCAM) [6]. Increasing evidences have suggested that all the three subgroups’ MUC members are closely linked to cancer initiation and progression. For example, Ganguly et al. found that secretory MUC5AC promoted neoplastic progression by augmenting KLF4-mediated pancreatic cancer cell stemness [7]; Xu et al. indicated that MUC1 was overexpressed in NSCLC and silence of MUC1 alleviated paclitaxel resistance of NSCLC [8]; Gao et al. suggested that MUC12 enhanced RCC progression by regulating c-Jun/TGF-β signaling [9]; Tiemin et al. found that MUC13 facilitated progression of intrahepatic cholangiocarcinoma via EGFR/PI3K/AKT pathways [10]. Moreover, dysregulated MUCs are reported to serve as potential biomarkers in multiple tumor types. For instance, serum MUC3A is a potential diagnostic biomarker for extrahepatic cholangiocarcinoma [11]; high expression of MUC3A is associated with localized clear-cell renal cell carcinoma [12]; bone marrow MUC4 expression had significant prognostic value in acute myeloid leukaemia [13].

In this study, we firstly determined the mRNA and protein expression of MUCs, then assessed the diagnostic and prognostic values of MUCs, and finally explored the underlying upstream ncRNA action mechanism and downstream molecular mechanism of MUC14 in breast cancer. Collectively, we established a potential MUC14-related ncRNA-mRNA axis in breast cancer, which provides key clues for developing effective therapeutic targets and promising biomarkers in breast cancer.

## 2. Materials and Methods

### 2.1. starBase Analysis

starBase (http://starbase.sysu.edu.cn/, accessed on 9 July 2021), an open-source platform for studying the miRNA-ncRNA, miRNA-mRNA, ncRNA-RNA, RNA-RNA, RBP-ncRNA and RBP-mRNA interactions from CLIP-seq, degradome-seq and RNA-RNA interactome data, was used to perform expression analysis for gene, miRNA and lncRNA in breast cancer [14]. starBase was also used to conduct expression correlation analysis for miRNA-lncRNA and lncRNA-mRNA pairs in breast cancer.

### 2.2. GEPIA Analysis

GEPIA (http://gepia.cancer-pku.cn/, accessed on 9 July 2021) is a newly developed interactive web server for analyzing the RNA sequencing expression data of 9736 tumors and 8587 normal samples from the TCGA and the GTEx projects, which was used to determine the mRNA expression of MUCs in breast cancer [15].

### 2.3. HPA Analysis

The protein expression of MUCs in breast cancer was analyzed using HPA database (http://www.proteinatlas.org/, accessed on 9 July 2021), which is a proteomic resource for biomarker discovery [16].

### 2.4. UALCAN Analysis

UALCAN (http://ualcan.path.uab.edu/index.html, accessed on 9 July 2021), a comprehensive, user-friendly and interactive web resource for analyzing cancer OMICS data, was also used to detect the protein expression levels of MUCs in breast cancer [17].

### 2.5. ROC Curve Analysis

As we previously described, the diagnostic values of MUCs in breast cancer were assessed by ROC curve analysis using TCGA breast cancer and normal breast tissues data with the help of GraphPad Prism software [5].

### 2.6. Kaplan-Meier Plotter Analysis

Kaplan-Meier plotter database (http://kmplot.com/analysis, accessed on 9 July 2021) is capable to access the effects of 54,000 genes on survival in 21 cancer types, including breast cancer, which was employed to perform survival analysis for MUCs, miRNAs, lncRNAs and target genes in breast cancer [18].

### 2.7. bc-GenExMiner Analysis

bc-GenExMiner (http://bcgenex.ico.unicancer.fr, accessed on 9 July 2021), an easy-to use online platform for analyzing gene expression, prognosis and correlation in breast cancer, was employed to determine MUC14 expression in breast cancer based on various clinicopathological features as we previously described [19,20].

### 2.8. miRNA Prediction

The miRNAs that could potentially bind to MUC14 were predicted using seven target gene prediction programs, including PITA, RNA22, miRmap, microT, miRanda, PicTar and TargetScan. Only those miRNAs appeared in more than 2 programs were included in our study.

### 2.9. miRNet Analysis

miRNet (http://www.mirnet.ca, accessed on 9 July 2021), a miRNA-centric network visual analytics platform, was used to predicted upstream lncRNAs of miR-137 or miR-429 [21]. Besides, miRNet was also introduced to predict the downstream target genes of MUC14-miR-137/miR-429 axis.

## 3. Results

### 3.1. The Expression of MUCs in Breast Cancer

**Figure 1:**
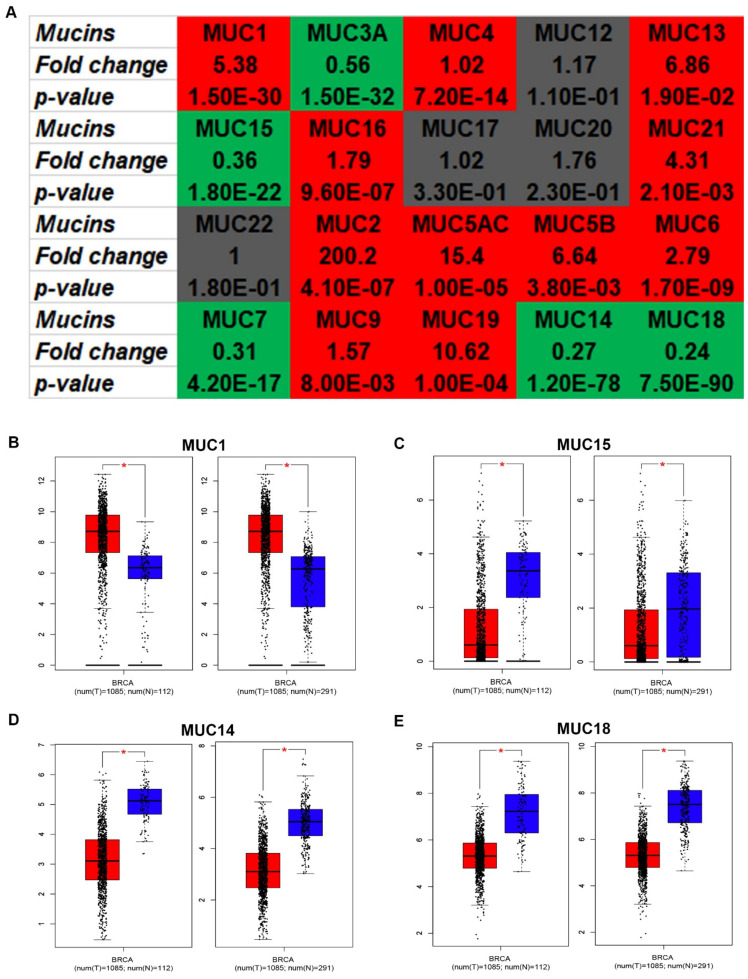
The mRNA expression of MUCs in breast cancer. (**A**) The mRNA expression landscape of MUCs in breast cancer determined by starBase. Red: high expression; green: low expression; grey: no statistical difference. The expression of MUC1 (**B**), MUC15 (**C**), MUC14 (**D**) and MUC18 (**E**) in breast cancer determined by GEPIA. * *p*-value < 0.05.

**Figure 2:**
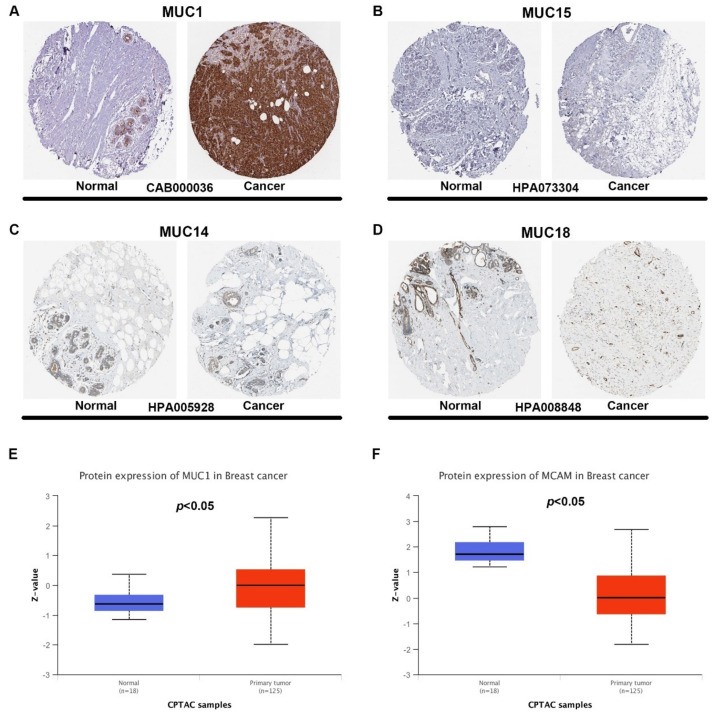
The protein expression of MUCs in breast cancer. The protein expression of MUC1 ((**A**), antibody: CAB000036), MUC15 ((**B**), antibody: HPA073304), MUC14 ((**C**), antibody: HPA005928) and MUC18 ((**D**), antibody: HPA008848) in breast cancer and normal control determined by HPA. The protein expression of MUC1 (**E**) and MUC14 (**F**) in breast cancer and normal control determined by CPTAC. “*p* < 0.05” was considered as statistically significant.

In this study, we firstly determined the mRNA expression of 20 MUCs in breast cancer using starBase. As shown in Figure 1A, 11 of 20 MUCs, including MUC1, MUC4, MUC13, MUC16, MUC21, MUC2, MUC5AC, MUC5B, MUC6, MUC9 and MUC19, were significantly upregulated in breast cancer tissues when compared with normal breast cancer tissues, and 5 of 20 MUCs, involving MUC3A, MUC15, MUC7, MUC14 and MUC18, were markedly downregulated in breast cancer samples. For MUC12, MUC17, MUC20 and MUC22, no statistical differences between cancer samples and normal controls were observed. Next, to validate these analytic results, another database, namely GEPIA, was introduced to further assess the mRNA expression levels of MUCs in breast cancer. Two types of control, containing “Match TCGA normal data” and “Match TCGA normal and GTEx data”, were employed. After performing differential expression analysis, only MUC1 (Figure 1B), MUC15 (Figure 1C), MUC14 (Figure 1D) and MUC18 (Figure 1E) were commonly dysregulated in the two analytic models. Moreover, the expression differences of MUCs among various major stage in breast cancer were also assessed using GEPIA database, and the results showed that only MUC16 and MUC14 presented statistical significance (Table S1). Intriguingly, these data were in accordance with the analytic results from starBase database. Subsequently, the protein expression levels of the four candidate MUCs in breast cancer were firstly analyzed using immunostaining results from HPA database. As presented in Figure 2A–D, MUC1 expression in breast cancer tissues was higher than that in normal breast tissues while MUC15, MUC14 and MUC18 were downregulated in cancer samples compared with normal controls. Furthermore, CPTAC was also utilized to detect the protein expression of the four MUCs in breast cancer. Identical with the results from HPA database, MUC1 expression was significantly upregulated while MUC18 (MCAM) expression was obviously downregulated in breast cancer samples (Figure 2E–F). However, no related protein expression data of MUC15 and MUC14 in breast cancer were obtained. Taken together, MUC1, MUC15, MUC14 and MUC18 were identified as the most potential MUC members in breast cancer and were selected for subsequent analysis.

### 3.2. The Diagnostic and Prognostic Values of Candidate MUCs in Breast Cancer

**Figure 3:**
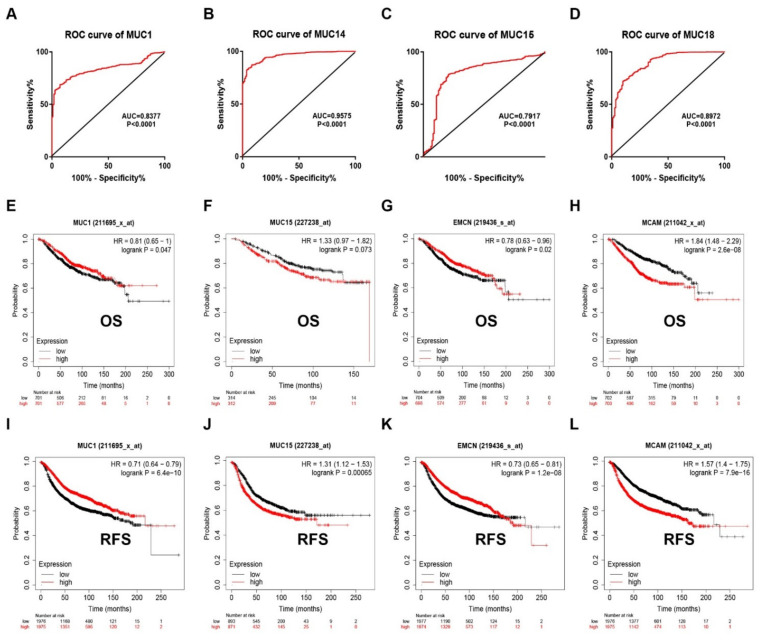
The diagnostic and prognostic values of MUC1, MUC14, MUC15 and MUC18 in breast cancer. (**A**) The ROC curve of MUC1 (**A**), MUC14 (**B**), MUC15 (**C**) and MUC18 (**D**) in breast cancer. The OS curve of MUC1 (**E**), MUC15 (**F**), MUC14 (**G**) and MUC18 (**H**) in breast cancer. The RFS curve of MUC1 (**I**), MUC15 (**J**), MUC14 (**K**) and MUC18 (**L**) in breast cancer. “*p* < 0.05” was considered as statistically significant.

Considering the expression change of MUC1, MUC15, MUC14 and MUC18 in breast cancer, we intended to ascertain if they possessed promising predictive roles for diagnosis and prognosis of breast cancer. Firstly, the diagnostic values of the four MUCs in breast cancer were evaluated through ROC curve analysis. As suggested in Figure 3A–D, all the four MUCs had the statistical abilities to distinguish breast cancer from normal controls. Next, Kaplan-Meier plotter was employed to assess the prognostic roles of MUC1, MUC15, MUC14 and MUC18 in breast cancer. Two indices, including overall survival (OS) and relapse free survival (RFS), were selected in this part. As presented in Figure 3E–H, breast cancer patients with higher expression of MUC1 and MUC14 but with lower expression of MUC15 and MUC18 had better OS. In accordance with OS analysis, breast cancer patients with increased expression of MUC1 and MUC14 but decreased expression of MUC15 and MUC18 possessed favorable RFS (Figure 3I–L). All these findings suggested that all the four MUCs might serve as potential diagnostic and prognostic biomarkers in breast cancer.

### 3.3. Identification of MUC14 as the Most Potential Protective Regulator in Breast Cancer

**Figure 4:**
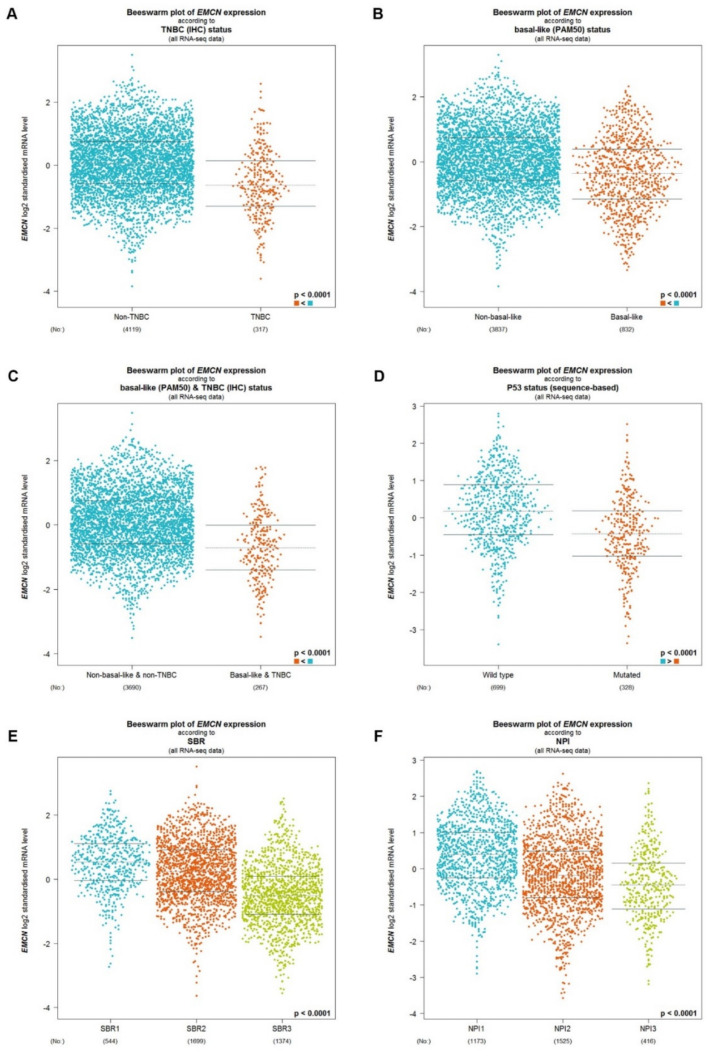
Expression differences of MUC14 in breast cancer based on different clinicopathological features. (**A**) MUC14 expression in non-TNBC and TNBC. (**B**) MUC14 expression in non-basal-like and basal-like breast cancer. (**C**) MUC14 expression in non-basal-like breast cancer & non-TNBC and basal-like breast cancer & TNBC. (**D**) MUC14 expression in P53 wild type and mutated breast cancer. (**E**) MUC14 expression in breast cancer based on various SBR grade. (**F**) MUC14 expression in breast cancer based on various NPI score. “*p* < 0.05” was considered as statistically significant.

By combination of expression analysis, ROC curve analysis and survival analysis, MUC14 was identified as the most promising MUC member in breast cancer, with decreased expression in breast cancer, potential diagnostic biomarker and favorable prognostic predictor. Then, bc-GenExMiner was utilized to further analyze if MUC14 functioned as a tumor suppressor in breast cancer. As shown in Figure 4A, MUC14 was significantly downregulated in TNBC compared with non-TNBC. MUC14 expression in basal-like breast cancer was also markedly lower than that in non-basal-like breast cancer (Figure 4B). Moreover, the expression of MUC14 in basal-like & triple-negative breast cancer was obviously decreased compared with non-basal-like & triple-negative breast cancer (Figure 4C). As presented in Figure 4D, MUC14 expression in P53 mutated breast cancer was statistically downregulated when compared with P53 wild type breast cancer. Furthermore, MUC14 expression was significantly negatively correlated with SBR grade (Figure 4E) and NPI score (Figure 4F). These findings suggested that MUC14 was negatively associated with malignant state of breast cancer and might act as a protective regulator in carcinogenesis and progression of breast cancer.

### 3.4. Prediction and Analysis of Upstream miRNAs of MUC14 in Breast Cancer

**Figure 5:**
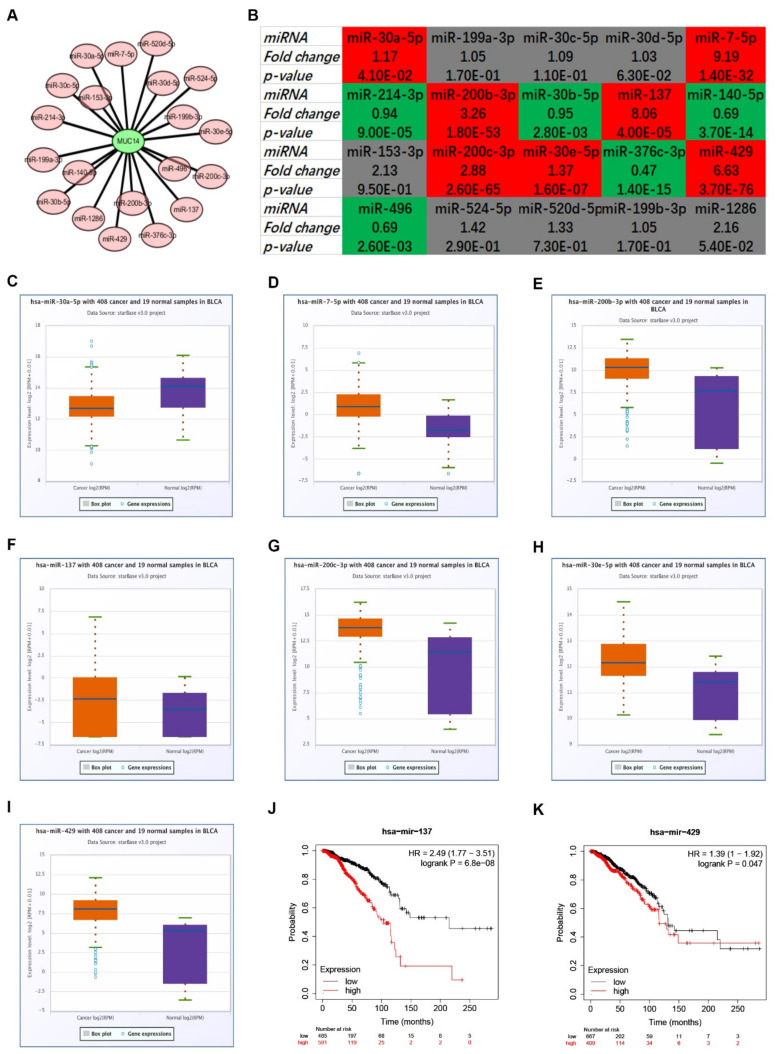
The potential upstream miRNAs of MUC14 in breast cancer. (**A**) A MUC14-miRNA regulatory network. (**B**) The expression landscape of 20 predicted miRNAs of MUC14 in breast cancer. The expression of miR-30a-5p (**C**), miR-7-5p (**D**), miR-200b-3p (**E**), miR-137 (**F**), miR-200c-3p (**G**), miR-30e-5p (**H**) and miR-429 (**I**) in breast cancer. (**J**) The prognostic value of miR-137 in breast cancer. (**K**) The prognostic value of miR-429 in breast cancer. “*p* < 0.05” was considered as statistically significant.

7 target gene prediction programs, including PITA, RNA22, miRmap, microT, miRanda, PicTar and TargetScan, were used to predict upstream miRNAs that could potentially bind to MUC14. Only those MUC14-miRNA pairs predicted by more than two prediction programs were included in our subsequent investigation. Consequently, 20 miRNAs were finally found. To better visualization, a MUC14-miRNA regulatory network was established as shown in Figure 5A. Based on the action mechanism of miRNA, there should be negative relationship between MUC14 and its potential upstream miRNAs. Therefore, the expression levels of the 20 miRNAs in breast cancer were firstly determined (Figure 5B). Among the 20 miRNAs, 7 and 5 miRNAs were significantly upregulated and downregulated in breast cancer compared with normal controls, respectively. The other 8 miRNAs were not statistically differentially expressed between normal breast tissues and breast cancer tissues. The 7 upregulated miRNAs, containing miR-30a-5p, miR-7-5p, miR-200b-3p, miR-137, miR-200c-3p, miR-30e-5p and miR-429, were presented in Figure 5C–I. Additionally, the prognostic values of the 7 miRNAs in breast cancer were also determined by Kaplan-Meier plotter. Only breast cancer patients with high expression of miR-137 (Figure 5J) or miR-429 (Figure 5K) indicated unfavorable prognosis. Taken all these findings into consideration, miR-137 and miR-429 might be two most potential upstream binding miRNAs of MUC14 in breast cancer.

### 3.5. Prediction and Analysis of Upstream lncRNAs of MUC14-miR-137/miR-429 Axis in Breast Cancer

**Figure 6:**
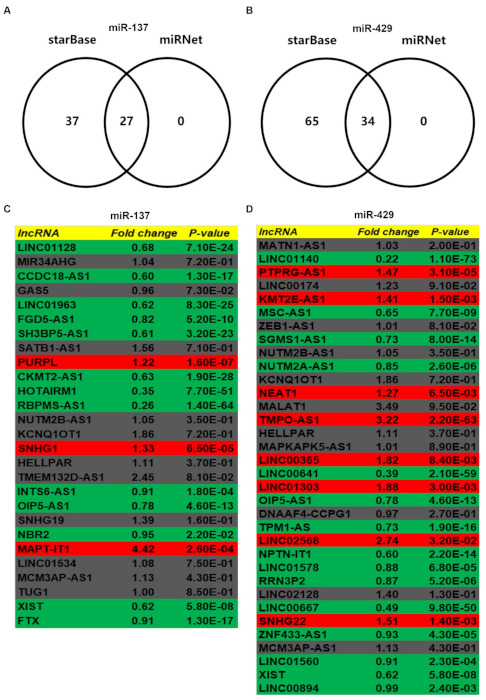
Prediction and expression analysis for upstream lncRNAs of miR-137 or miR-429 in breast cancer. (**A**) The intersection analysis of predicted lncRNAs from starBase and miRNet. (**B**) The intersection analysis of predicted lncRNAs from starBase and miRNet. (**C**) The expression landscape of predicted lncRNAs of miR-137 in breast cancer. (**D**) The expression landscape of predicted lncRNAs of miR-429 in breast cancer. “*p* < 0.05” was considered as statistically significant.

**Figure 7:**
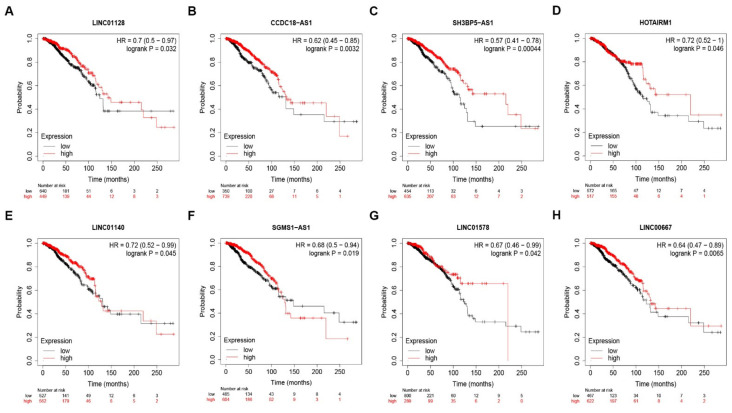
Survival analysis for potential lncRNAs of miR-137 or miR-429 in breast cancer. (**A**) The prognostic value of LINC01128 (**A**), CCDC18-AS1 (**B**), SH3BP5-AS1 (**C**), HOTAIRM1 (**D**), LINC01140 (**E**), SGMS1-AS1 (**F**), LINC01578 (**G**) and LINC00667 (**H**) in breast cancer. “*p* < 0.05” was considered as statistically significant.

**Figure 8:**
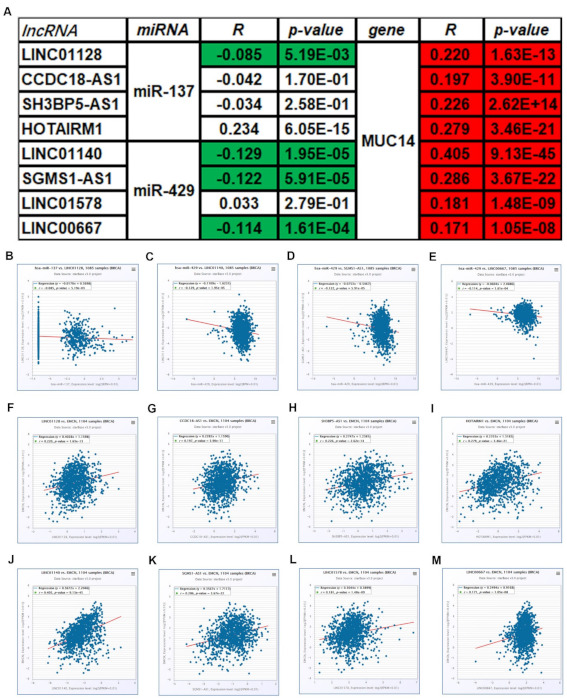
Expression correlation analysis for lncRNA-miRNA or lncRNA-MUC14 pairs in breast cancer. (**A**) The correlation landscape of RNA-RNA pairs in breast cancer. The expression correlation of LINC01128/miR-137 (**B**), LNC01140/miR-429 (**C**), SGMS1-AS1/miR-429 (**D**), LINC00667/miR-429 (**E**)**,** LINC01128/MUC14 (**F**), CCDC18-AS1/MUC14 (**G**), SH3BP5-AS1/MUC14 (**H**), HOTAIRM1/MUC14 (**I**), LINC01140/MUC14 (**J**), SGMS1-AS1/MUC14 (**K**), LINC01578/MUC14 (**L**) and LINC00667/MUC14 (**M**) in breast cancer. “*p* < 0.05” was considered as statistically significant.

To further explore the upstream molecular mechanism of MUC14-miR-137/miR-429 axis in breast cancer, starBase and miRNet were used to predict those lncRNAs that could potentially bind to miR-137 or miR-429. As suggested in Figure 6A and Figure 6B, 27 and 24 lncRNAs were commonly appeared in the two prediction lncRNA sets, respectively. According to ceRNA hypothesis, the potential upstream lncRNAs of MUC14-miR-137/miR-429 in breast cancer should act as tumor suppressive lncRNAs in breast cancer. First of all, the expression levels of predicted lncRNAs of miR-137 or miR-429 were analyzed using starBase (Figure 6C,D). Then, survival analysis for those downregulated lncRNAs was performed. As shown in Figure 7, only high expression of LINC01128, CCDC18-AS1, SH3BP5-AS1, HOTAIRM1, LINC01140, SGMS1-AS1, LINC01578 or LINC00667 had favorable prognosis in breast cancer. Subsequently, the expression correlation of lncRNA with miRNA or MUC14 was determined using TCGA breast cancer data (Figure 8A). As presented in Figure 8B–M, there were four lncRNA-miRNA pairs (LINC01128/miR-137, LINC01140/miR-429, SGMS1-AS1/miR-429 and LINC00667/miR-429) with negative expression correlation and eight lncRNA-MUC14 pairs (LINC01128/MUC14, CCDC18-AS1/MUC14, SH3BP5-AS1/MUC14, HOTAIRM1/MUC14, LINC01140/MUC14, SGMS1-AS1/MUC14, LINC01578/MUC14 and LINC00667/MUC14) with positive expression correlation in breast cancer.

### 3.6. Identification of Potential Downstream Target Genes of MUC14-miR-137/miR-429 Axis in Breast Cancer

**Figure 9:**
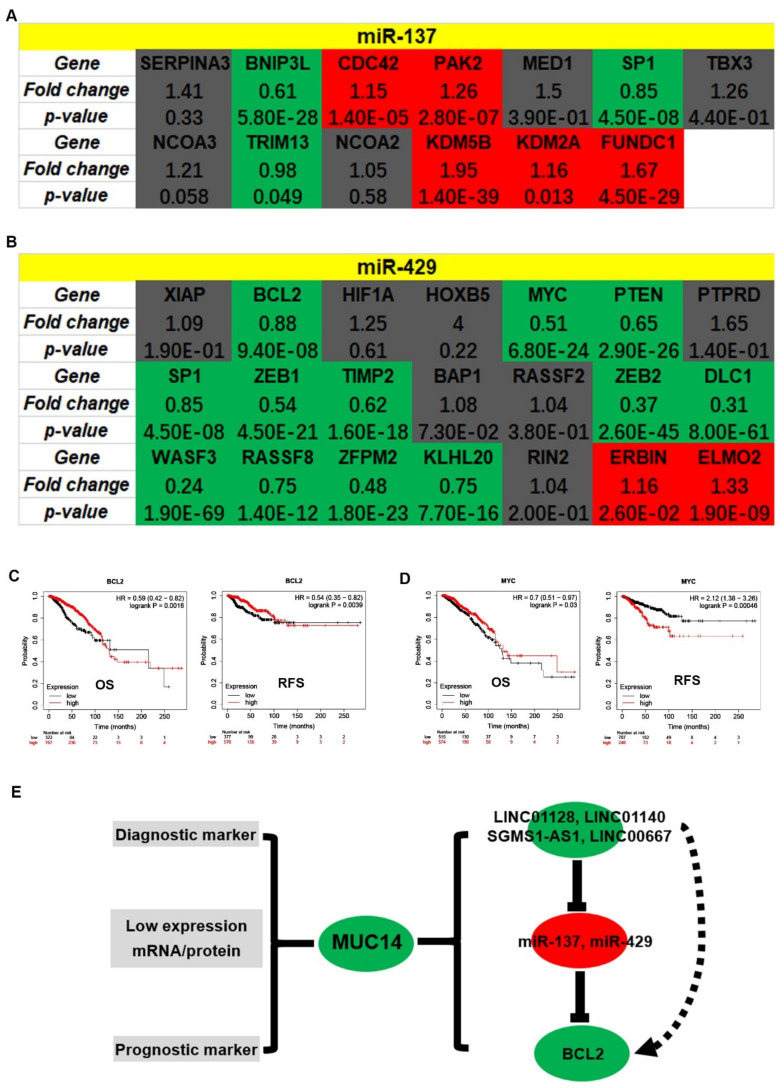
Identification of potential downstream targets of MUC14-miR-137/miR-429 axis in breast cancer. (**A**) The expression landscape of target genes of miR-137 (**A**) and miR-429 (**B**) in breast cancer. Red: high expression; green: low expression; grey: no statistical difference. The prognostic values of BCL2 (**C**) and MYC (**D**) in breast cancer. (**E**) The mechanism graph of the MUC14-related ncRNA-mRNA axis in breast cancer. “*p* < 0.05” was considered as statistically significant.

Subsequently, we predicted downstream target genes of miR-137 or miR-429 using miRNet. To improve the accuracy, only those target genes validated by reporter assay, qRT-PCR or western blot were included, and 50 and 39 target genes were predicted to bind to miR-137 and miR-429, respectively. The expression relationships between miRNAs and their corresponding target genes in breast cancer were assessed by usage of TCGA breast cancer data. As listed in Table 1 and Table 2, miR-137 and miR-429 were significantly negatively correlated with 13 and 21 target genes, respectively. Then, the expression levels of these target genes of miR-137 and miR-429 were determined in breast cancer. As shown in Figure 9A,B, 3 of 13 and 12 of 21 target genes were markedly downregulated in breast cancer tissues compared with normal breast tissues. Next, the prognostic values of these downregulated target genes in breast cancer were also evaluated by Kaplan-Meier plotter. As a result, increased expression of BCL2 indicated favorable OS and RFS (Figure 9C) and high expression of MYC had good OS but poor RFS in breast cancer (Figure 9D). Thus, BCL2 might be the most potential downstream target of MUC14-miR-137/miR-429 axis in breast cancer. Collectively, we established a potential ncRNA-mRNA axis, which might be involved in MUC14′s roles in breast cancer (Figure 9E).

## 4. Discussion

Growing evidences have shown that MUCs are frequently aberrantly expressed in multiple malignancies and their dysregulation is closely correlated with initiation and progression of malignancies and can serve as biomarkers for diagnosis and prognosis of breast cancer. In this study, we aimed to comprehensively assess expression, diagnosis and prognosis of MUCs in breast cancer. Besides, it has been widely acknowledged that genetic and epigenetic alterations account for cancer pathogenesis. Thus, we also explored the upstream and downstream molecular mechanisms of MUCs in breast cancer.

By combination of expression, diagnosis and prognosis analyses, MUC14 was identified as the most potential member among MUCs in breast cancer. In our study, MUC14 was significantly downregulated in breast cancer, possessed statistical ability to distinguish breast cancer tissues from normal breast tissues and its downregulation indicated poor prognosis in breast cancer. Previous investigations also showed that MUC14 was a prognostic biomarker for several types of human cancer, including hepatocellular carcinoma [22], gastric cancer [23], diffuse type gastric cancer [24] and clear cell renal cell carcinoma [25]. To date, no research regarding expression, diagnosis and prognosis of MUC14 in breast cancer has been reported. MUC14 expression was markedly decreased in breast cancer with specific clinicopathological characteristics, such as TNBC, basal-like breast cancer, P53 mutated breast cancer, advanced SBR grade and NPI score, further suggesting that MUC14 might act as a tumor suppressor in breast cancer.

miRNA play key roles in a series of biological and pathological processes via suppressing gene expression by binding to 3′UTR of mRNAs [26,27,28]. Therefore, the upstream miRNAs of MUC14 were predicted using several target gene prediction programs. After performing expression analysis and survival analysis for these miRNAs, two potential oncogenic miRNAs, including miR-137 and miIR-429, were identified in breast cancer. Numerous studies have suggested that miR-137 and miR-429 were linked to occurrence and progression of breast cancer [29,30,31].

In 2011, Leonardo et al. proposed that noncoding RNA and messenger RNAs could “talk” to each other using microRNA response elements (MREs) [32]. Thus, we further predicted the binding lncRNAs of miIR-137 and miR-429 using two online databases, namely starBase and miRNet. By a series of in silico analyses, including expression analysis, survival analysis and correlation analysis, five potential lncRNA-miRNA pairs, containing LINC01128/miR-137, LINC01140/miR-429, SGMS1-AS1/miR-429 and LINC00667/miR-429, were identified. Among these lncRNA-miRNA pairs, LINC00667/miR-429 pair has been reported to be involved in the vasculogenic mimicry of glioma cells [33]. Moreover, some of lncRNAs mentioned in these lncRNA-miRNA pairs have been found to be associated with tumorigenesis of breast cancer. For example, Li et al. indicated that LINC01140 downregulation was correlated with adverse features of breast cancer [34].

To further understand the possible molecular action mechanism of MUC14, the downstream target genes of miR-137 and miR-429 were predicted. In order to improve the accuracy of prediction, only miRNA-target gene pairs proved by reporter assay, qRT-PCR and western blot were included for subsequent analyses. After performing correlation analysis, expression analysis and survival analysis, BCL2 was identified as the most potential downstream target gene of MUC14-miR-137/miR-429 axis in breast cancer. BCL2 is an integral outer mitochondrial membrane protein that blocks the apoptotic death of some cells such as lymphocytes [35,36]. Increasing studies have demonstrated that BCL2 is involved in carcinogenesis of multiple types of cancer, including breast cancer [37,38,39]. Taken together, BCL2 might be the most potential target involved in the constructed MUC14-related ncRNA-mRNA network in breast cancer.

Despite a key MUC14-associated ncRNA-mRNA network in breast carcinogenesis has been established, there were some limitations in this work. For example, these findings were concluded based on online public data, including expression and survival data. No experimental validation, including cell line, clinical samples or functional assay, was employed in this study. In the future, much more basic experiments and clinical trials should be conducted to verify these findings.

## 5. Conclusions

Collectively, we established a potential MUC14-related ncRNA-mRNA network, in which all RNAs were significantly differentially expressed between breast cancer and normal breast samples and also possessed significant prognostic values in breast cancer. This network provided important clues for exploring a comprehensive molecular explanation of carcinogenesis of breast cancer and developing promising therapeutic targets and biomarkers in breast cancer. However, these findings should be confirmed by much more experimental assays in the future.

## Figures and Tables

**Table 1:** The expression correlation of miR-137 with its predicted binding target genes in breast cancer determined by starBase.

ID	Target	R	P
**miR-137**	**SERPINA3**	**−0.061**	**4.37 × 10^−2^**
miR-137	AKT2	−0.029	3.37 × 10^−1^
miR-137	BMP7	0.089	3.30 × 10^−3^
**miR-137**	**BNIP3L**	**−0.080**	**8.31 × 10^−3^**
**miR-137**	**CDC42**	**−0.154**	**3.52 × 10^−7^**
miR-137	CDK6	0.230	1.94 × 10^−14^
miR-137	CSE1L	−0.043	1.57 × 10^−1^
miR-137	CTBP1	0.066	3.06 × 10^−2^
miR-137	E2F6	−0.047	1.22 × 10^−1^
miR-137	EGFR	0.216	6.73 × 10^−13^
miR-137	ESRRA	0.171	1.49 × 10^−8^
miR-137	EZH2	0.100	9.51 × 10^−4^
miR-137	KIT	0.029	3.34 × 10^−1^
miR-137	MET	0.204	1.32 × 10^−11^
miR-137	MITF	0.005	8.67 × 10^−1^
miR-137	MSI1	0.073	1.66 × 10^−2^
miR-137	COX2	0.000	1.00
miR-137	MYO1C	−0.034	2.59 × 10^−1^
miR-137	YBX1	0.215	8.68 × 10^−13^
**miR-137**	**PAK2**	**−0.118**	**1.04 × 10^−4^**
**miR-137**	**MED1**	**−0.095**	**1.82 × 10^−3^**
miR-137	PRKAA1	0.058	5.83 × 10^−2^
miR-137	PTGS2	0.131	1.44 × 10^−5^
miR-137	PXN	0.049	1.04 × 10^−1^
miR-137	RASGRF1	0.130	1.74 × 10^−5^
miR-137	RORA	−0.059	5.21 × 10^−2^
miR-137	CXCL12	−0.023	4.52 × 10^−1^
miR-137	SLC6A3	0.108	3.55 × 10^−4^
**miR-137**	**SP1**	**−0.201**	**2.25 × 10^−11^**
**miR-137**	**TBX3**	**−0.196**	**8.10 × 10^−11^**
miR-137	TGFB2	0.095	1.71 × 10^−3^
**miR-137**	**NCOA3**	**−0.121**	**6.42 × 10^−5^**
miR-137	CUL4A	−0.010	7.33 × 10^−1^
miR-137	DCLK1	−0.055	7.22 × 10^−2^
miR-137	KLF4	−0.005	8.77 × 10^−1^
miR-137	KDM4A	0.029	3.40 × 10^−1^
**miR-137**	**TRIM13**	**−0.094**	**1.98 × 10^−3^**
**miR-137**	**NCOA2**	**−0.142**	**2.86 × 10^−6^**
**miR-137**	**KDM5B**	**−0.124**	**3.95 × 10^−5^**
miR-137	GLIPR1	0.139	4.31 × 10^−6^
miR-137	KLF12	−0.002	9.55 × 10^−1^
miR-137	Nr1i3	0.100	9.75 × 10^−4^
**miR-137**	**KDM2A**	**−0.121**	**6.52 × 10^−5^**
miR-137	KDM1A	0.037	2.23 × 10^−1^
miR-137	KDM7A	−0.020	5.20 × 10^−1^
miR-137	CALN1	0.048	1.18 × 10^−1^
miR-137	ZNF804A	0.010	7.44 × 10^−1^
miR-137	MTDH	−0.045	1.35 × 10^−1^
miR-137	FMNL2	0.158	1.83 × 10^−7^
**miR-137**	**FUNDC1**	**−0.127**	**2.63 × 10^−5^**

**Table 2:** The expression correlation of miR-429 with its predicted binding target genes in breast cancer determined by starBase.

ID	Target	R	P
**miR-429**	**XIAP**	**−0.069**	**2.31 × 10^−2^**
miR-429	RERE	0.062	4.10 × 10^−2^
**miR-429**	**BCL2**	**−0.071**	**1.98 × 10^−2^**
miR-429	CRKL	−0.022	4.65 × 10^−1^
miR-429	DNMT1	0.079	9.56 × 10^−3^
miR-429	EZH2	0.215	8.50 × 10^−13^
**miR-429**	**HIF1A**	**−0.072**	**1.77 × 10^−2^**
**miR-429**	**HOXB5**	**−0.082**	**6.84 × 10^−3^**
miR-429	IL4	−0.03	3.28 × 10^−1^
miR-429	MYB	0.04	1.87 × 10^−1^
**miR-429**	**MYC**	**−0.066**	**3.06 × 10^−2^**
**miR-429**	**PTEN**	**−0.165**	**4.99 × 10^−8^**
**miR-429**	**PTPRD**	**−0.213**	**1.24 × 10^−12^**
miR-429	RBBP4	0.194	1.16 × 10^−10^
miR-429	SHC1	−0.153	4.32 × 10^−7^
miR-429	FSCN1	−0.054	7.76 × 10^−2^
miR-429	SOX2	−0.005	8.63 × 10^−1^
**miR-429**	**SP1**	**−0.068**	**2.48 × 10^−2^**
**miR-429**	**ZEB1**	**−0.316**	**1.38 × 10^−26^**
**miR-429**	**TIMP2**	**−0.308**	**2.73 × 10^−25^**
miR-429	VEGFA	0.058	5.59 × 10^−2^
**miR-429**	**BAP1**	**−0.104**	**6.18 × 10^−4^**
miR-429	KLF11	−0.056	6.35 × 10^−2^
miR-429	ONECUT2	0.01	7.51 × 10^−1^
**miR-429**	**RASSF2**	**−0.199**	**3.83 × 10^−11^**
**miR-429**	**ZEB2**	**−0.306**	**6.76 × 10^−25^**
**miR-429**	**DLC1**	**−0.283**	**2.18 × 10^−21^**
**miR-429**	**WASF3**	**−0.145**	**1.58 × 10^−6^**
**miR-429**	**RASSF8**	**−0.144**	**1.77 × 10^−6^**
miR-429	WDR37	0.046	1.33 × 10^−1^
**miR-429**	**ZFPM2**	**−0.291**	**1.18 × 10^−22^**
miR-429	OSTF1	0.051	9.35 × 10^−2^
**miR-429**	**KLHL20**	**−0.201**	**2.61 × 10^−11^**
**miR-429**	**RIN2**	**−0.175**	**6.67 × 10^−9^**
miR-429	VAC14	0.053	8.28 × 10^−2^
**miR-429**	**ERBIN**	**−0.151**	**5.96 × 10^−7^**
**miR-429**	**ELMO2**	**−0.092**	**2.29 × 10^−3^**
miR-429	TCF7L1	−0.016	5.98 × 10^−1^
miR-429	MALAT1	−0.02	5.03 × 10^−1^

## Data Availability

All the data have been presented in the manuscript. Table S1 also published in your online website.

## Supplementary Materials

The following are available online at https://www.mdpi.com/article/10.3390/genes12111677/s1, Table S1: The expression differences of MUCs family among various major pathological stage in breast cancer using TCGA data.
