# Supplementary material for: MUC14-Related ncRNA-mRNA Network in Breast Cancer
Source: Genes (Basel). 2021 Oct 23;12(11):1677. doi: 10.3390/genes12111677 (PMC8620399; doi:10.3390/genes12111677)
Supplement: Supplementary file 1 [file genes-12-01677-s001.zip › genes-1400532-supplementary.pdf]

Table S1. The expression differences of MUCs family among various major pathological stage in breast cancer using TCGA data.

Gene name	F-value	P-value
MUC1	1.610	1.70E-01
MUC3A	1.140	3.38E-01
MUC4	0.637	6.36E-01
MUC12	1.390	2.36E-01
MUC13	0.603	6.61E-01
MUC15	0.921	4.51E-01
**MUC16**	**3.210**	**1.25E-02**
MUC17	0.980	4.17E-01
MUC20	0.952	4.33E-01
MUC21	0.361	8.37E-01
MUC22	0.290	8.85E-01
MUC2	0.323	8.63E-01
MUC5AC	0.284	8.89E-01
MUC5B	1.540	1.88E-01
MUC6	0.997	4.08E-01
MUC7	1.710	1.46E-01
MUC9	0.101	9.82E-01
MUC19	0.972	4.22E-01
**MUC14**	**5.830**	**1.21E-04**
MUC18	0.269	8.98E-01
